# Influences of the three-dimensional parameters of pterygium on corneal astigmatism and the intraocular lens power calculation

**DOI:** 10.1038/s41598-020-61959-3

**Published:** 2020-03-19

**Authors:** Yating Tang, Dongjin Qian, Ling Wei, Yu Du, Xiaodi Qiu, Yi Lu, Xiangjia Zhu

**Affiliations:** 1 Department of Ophthalmology and Eye Institute, Eye and ENT Hospital of Fudan University, Shanghai, 200031 China; 2 NHC Key Laboratory of Myopia (Fudan University), Key Laboratory of Myopia, Chinese Academy of Medical Science; and Shanghai Key Laboratory of Visual Impairment and Restoration, Shanghai, 200031 China

**Keywords:** Corneal diseases, Lens diseases

## Abstract

Pterygium morphology had great effect on corneal astigmatism and intraocular lens (IOL) power calculation in cataract patients. However, previous studies all focused on the pterygium surface parameters, the invasion degree or cross-sectional area of the pterygia into the corneal stroma were neglected. We studied the effect of three-dimensional parameters of pterygium on corneal astigmatism and IOL power prediction. We enrolled 81 eyes of 81 patients with primary nasal pterygium, measured the corneal astigmatism (Pentacam HR) and predicted IOL power change (IOLmaster500) before and after pterygium surgery. The three-dimensional parameters of pterygium (length, width, area, height and invasion cross-sectional area) were measured by slit lamp photography and Scheimpflug images. After pterygium surgery, corneal astigmatism decreased from 4.35 ± 4.24 to 1.07 ± 0.95 D and total corneal refractive power increased from 43.02 ± 1.96 to 43.95 ± 0.95 D (both P < 0.001). The predicted IOL power decreased from 22.87 ± 2.82 to 21.71 ± 2.85 D (P < 0.001) after surgery. Notably, 34 eyes (41.98%) had ≥3.0 D of pterygium induced astigmatism (PIA), and 33 eyes (40.74%) had ≥1.0 D of predicted IOL power change. PIA was independently influenced by the pterygium surface area (r = 0.43, P < 0.001) and cross-sectional area (r = 1.25, P = 0.018), while the predicted IOL power change was independently affected by the pterygium width (r = 0.70, P < 0.001). Cataract surgeons could evaluate the effects of a pterygium according to its three-dimensional parameters and prepare an optimal surgical strategy for cataract combined pterygium patients.

## Introduction

Pterygium is a common ocular surface disease worldwide. It can occur concurrently with cataract because both diseases have major incidence among elderly people^1–4^. A large pterygium can induce significant corneal astigmatism and consequently affect the accuracy of the intraocular lens (IOL) power calculation^5,6^. Thus, cataract surgeons must understand the influences of pterygium on corneal astigmatism and the IOL power calculation before deciding on appropriate surgical strategies. Previous studies have indicated a significant correlation between pterygium-induced astigmatism (PIA) and its size, and the predicted IOL power would increase in eyes with a pterygium^5,7–11^.

However, these studies merely carried out two-dimensional analyses of pterygium morphology, including the width, length and area. No study has taken the degree or cross-sectional area of the pterygium invading the corneal stroma into consideration. When performing pterygium surgeries, surgeons can actually feel that pterygium with similar size of lesions might vary from one another with some are buried deeply into the corneal stroma and some are not. Thus, it seems that pterygium of the same surface size may have significantly different effects on the corneal astigmatism depending on their invasion degree or cross-sectional area in the stroma.

In the current study, we aimed to make a three-dimensional evaluation of pterygium (not only the width, length and area but also the height and invasion cross-sectional area) on its effects on corneal astigmatism and IOL power calculation.

## Patients and Methods

This study adhered to the tenets of the Declaration of Helsinki. Approval was obtained from the Human Ethics Committee of the Eye and ENT Hospital of Fudan University, and all patients signed a statement of informed consent before treatment and follow-up.

This prospective study included 81 eyes from 81 patients with a concurrent cataract and primary pterygium. All patients underwent pterygium excision and limbus conjunctival autograft surgery from March to September of 2018 in the Eye and Ear, Nose, Throat (ENT) Hospital of Fudan University, Shanghai. Although keratometry data have been reported to stabilize 1 week after pterygium surgery, all patients in this study were followed up for 1 month. The exclusion criteria were a recurrent or double-headed pterygium, prior trauma or surgery, corneal diseases other than pterygium that might affect the corneal regularity or biomechanics, keratitis, keratoconus, glaucoma, uveitis, severe fundus pathologies.

The pterygium excision and limbus conjunctival autograft surgery was conducted as follows. After subconjunctival injection of lidocaine-epinephrine into the pterygium tissue, the pterygium head was bluntly lifted off from the cornea and trimmed at its body from the rest approximately 4 mm from the corneal limbus. Subconjunctival tissue was removed, and the scarring corneal area was polished carefully. Then, a conjunctival autograft tissue was shifted from the superior bulbar conjunctiva to cover the defective area with a limbus-to-limbus overlap and fixed with 8-0 absorbable Vicryl sutures. Postoperatively, the topical antibiotic levofloxacin (Cravit, Santen, Japan), prednisolone acetate (Pred Forte, Allergan, Ireland) and artificial tears 0.3% sodium hyaluronate (Hialid, Santen, Japan) were used for 3 weeks.

**Figure 1:**
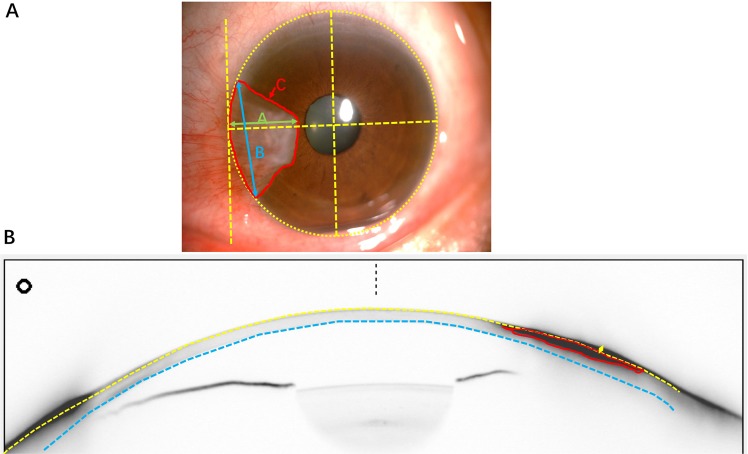
(Method) (**A**) A representative photograph illustrating measurement of the area (A, red area), length (**B**, green arrow) and width (**C**, blue arrow) of the pterygium region involving the cornea using ImageJ software. All photos were taken with the Topcon slit lamp photography system (SL-D7W, Japan) under ×16 magnification. The length, width and area were calculated using ImageJ software. (**B**) A representative Scheimpflug image illustrating measurement of the cross-sectional area of the pterygium (red line). We selected the Scheimpflug image of the horizontal meridian and adjusted the contrast of the image to clearly present the pterygium region. The yellow and blue arc lines represent the anterior and posterior surfaces of the cornea, and the red line represents the pterygium region. The yellow arrow represents the pterygium height (above the cornea). Then, the cross-sectional area of the pterygium was measured using ImageJ software.

An anterior segment photograph was taken with a slit lamp photography system (SL-D7W, Topcon, Japan) before the pterygium surgery. All images were taken under the same magnification (×16). The pterygium’s length, width and area were calculated using ImageJ software (Wayne Rasband, NIH, USA). The pterygium length was defined as a horizontal line from the corneal limbus to the pterygium head. The pterygium width was defined as the distance between both points of the pterygium growing into the corneal limbus, and the area was defined as the pterygium-covered cornea (Fig. 1A).

The corneal astigmatism, total corneal refractive power (TCRP) and pterygium cross-sectional area were measured using the Pentacam HR (Oculus Inc., Wetzlar, Germany) before and after pterygium surgery by the same doctor. For the cross-sectional area calculation, we selected the Scheimplug image at the horizontal meridian and adjusted the contrast of the image to present the pterygium region clearly. The cross-sectional area and height was calculated using ImageJ software (Fig. 1B). PIA was defined as the vector difference of topographic astigmatism before and 1 month after surgery (preoperatively minus postoperatively) according to a previous study.

The IOL power was calculated using the IOLmaster500 (Carl Zeiss Meditec, Germany) before and after pterygium surgery. Because the cornea was invaded by the pterygium, an accurate corneal curvature could not be obtained from many patients using the IOL master preoperatively. Therefore, we used the corneal curvature data from Pentacam to calculate the IOL power before and after pterygium surgery. We used the same IOL power calculation formula (SRK-T) for all patients. The predicted IOL power change was defined as the difference in the predicted IOL power before and 1 month after surgery (preoperatively minus postoperatively). All IOL power calculations were conducted by one experienced technician.

We analyzed the data using SPSS Statistics version 24.0 (IBM/SPSS, Inc., Chicago, IL, USA). Comparisons of data collected before and 1 month after surgery were performed with a paired t test. We carried out univariate Pearson correlation and linear regression analyses to analyze each influencing factor of PIA and prediction of the IOL power change. Stepwise linear regression (backward) was conducted to evaluate the independent predictive factors of PIA and the predicted IOL power change. Statistical significance was defined as P ≤ 0.05.

## Results

The baseline data from the included patients are presented in Table 1. The study included 32 males (39.51%) and 49 females (60.49%), and the mean age of the enrolled patients was 62.8 ± 8.76 years. The pterygium length and width were 2.75 ± 1.06 mm and 4.86 ± 0.94 mm, respectively. The pterygium surface area and cross-sectional area were 10.86 ± 5.36 mm^2^ and 0.93 ± 0.70 mm^2^, respectively.

**Table 1:** Demographic data for the enrolled patients.

Characteristics	Value	Range
Eye (Participates)	81 (81)	—
Gender (male, %)	32 (39.51)	—
Age (year) (mean ± SD)	62.80 ± 8.76	43–85
Right eye (%)	39 (48.15)	—
Left eye (%)	42 (51.85)	
Axial length (mm) (mean ± SD)	23.27 ± 0.96	21.22–28.39
Pterygium length (mm) (mean ± SD)	2.75 ± 1.06	0.85–5.67
Pterygium width (mm) (mean ± SD)	4.86 ± 0.94	2.05–6.80
Pterygium area (mm^2^) (mean ± SD)	10.86 ± 5.36	1.72–26.26
Pterygium cross-sectional area (mm^2^) (mean ± SD)	0.93 ± 0.70	0.13–2.98
Pterygium height (μm) (mean ± SD)	270.0 ± 87.14	90–450

**Figure 2:**
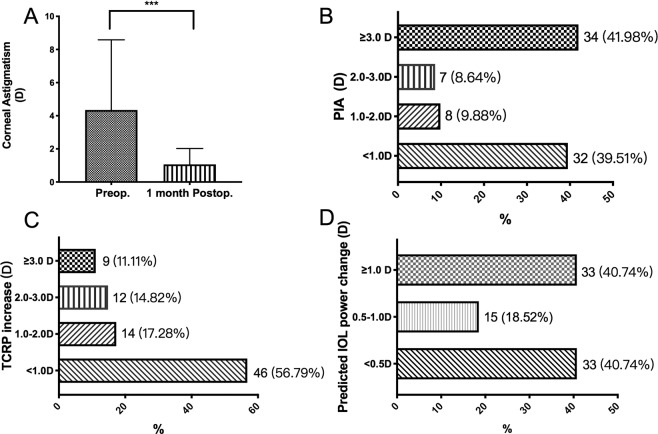
(**A**) Corneal astigmatism changes before and after pterygium surgery. Corneal astigmatism decreased significantly after pterygium surgery (***P < 0.001). (**B**) The pterygium-induced astigmatism (PIA) distribution (<1.0 D 39.51%, 1.0–2.0 D 9.88%, 2.0–3.0 D 8.64% and ≥3.0 D 41.98%). (**C)** The distribution of the TCRP increase (<1.0 D 56.79%, 1.0–2.0 D 17.28%, 2.0–3.0 D 14.82% and ≥3.0 D 11.11%). (**D)** The distribution of the predicted IOL power change (<0.5 D 40.74%, 0.5–1.0 D 18.52% and ≥1.0 D 40.74%).

At 1 month after surgery, the corneal astigmatism decreased from 4.35 ± 4.24 D to 1.07 ± 0.95 D (P < 0.001, Fig. 2A); a total of 32 eyes (39.51%) had PIA < 1.0 D, 8 eyes (9.88%) had PIA 1.0–2.0 D, 7 eyes (8.64%) had PIA 2.0–3.0 D and 34 eyes (41.98%) had PIA ≥ 3.0 D (Fig. 2B). The TCRP increased from 43.02 ± 1.96 D to 43.95 ± 0.95 D (P < 0.001) and 35 eyes (43.21%) had the TCRP increased ≥1.0 D (Fig. 2C). The predicted IOL power decreased from 22.87 ± 2.82 D to 21.71 ± 2.85 D (P < 0.001). A predicted IOL power change of <0.5 D was found in 33 eyes (40.74%), 0.5–1.0 D was found in 15 eyes (18.52%), and ≥1.0 D was found in 33 eyes (40.74%) (Fig. 2D).

**Figure 3:**
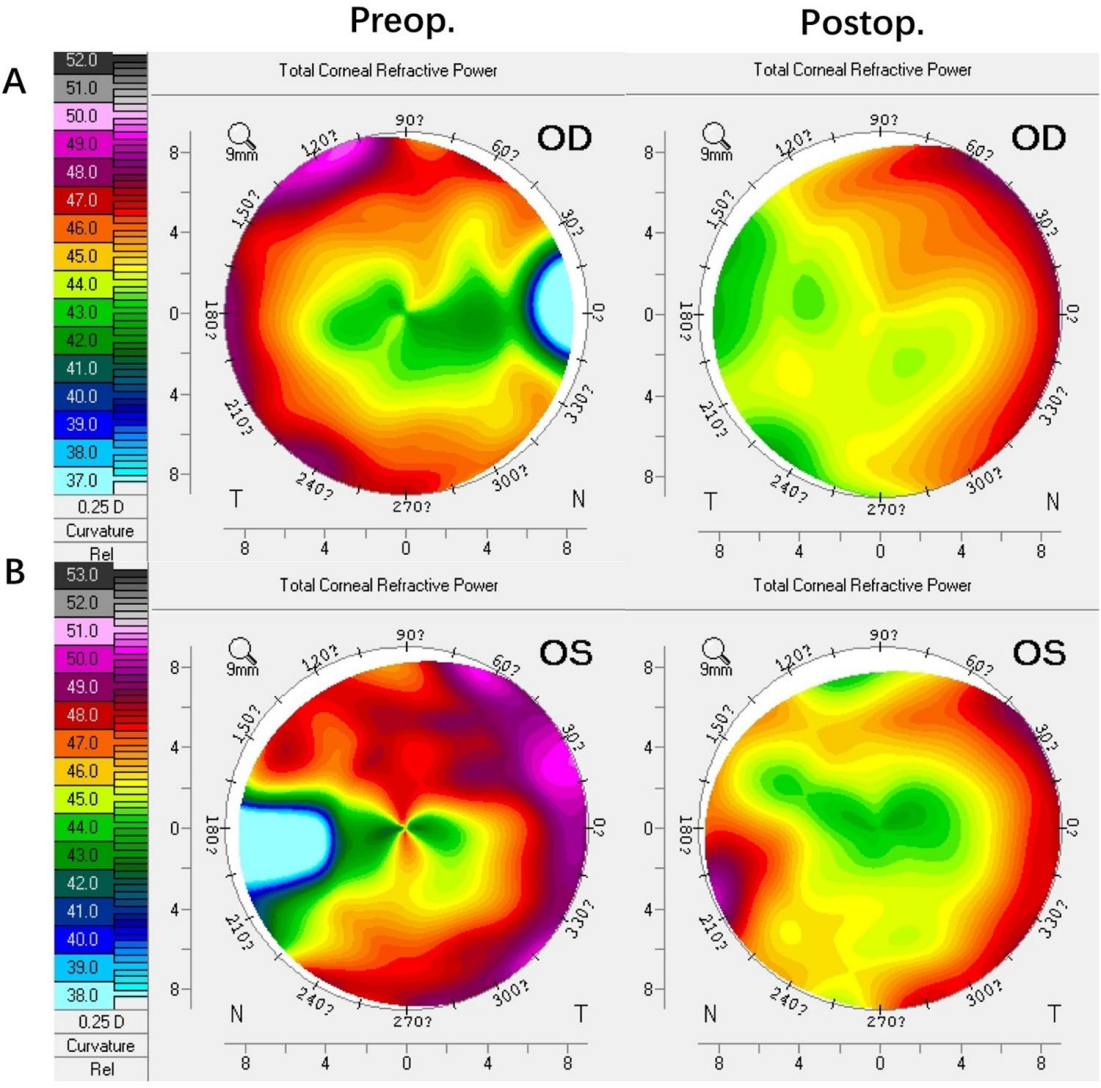
Representative total corneal refractive power maps of two pterygium cases before and after pterygium surgery. There was a tendency for a flattened to steep transition on the horizontal meridian.

We found a significant change in TCRP on the horizontal meridian from flattened to steeped tendency after the pterygium surgery. Figure 3 shows representative TCRP maps of two pterygium cases before and after pterygium surgery. The TCRP increased from 44.1 D to 44.9 D in case 1 (Fig. 3A) and from 45.5 D to 46.7 D in case 2 (Fig. 3B).

**Figure 4:**
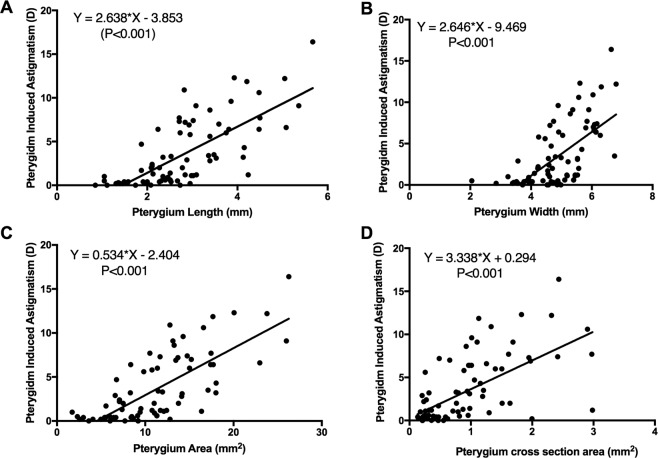
Scatter plots showing the univariate analysis results. Pearson correlation analysis showed significant correlations between PIA (pterygium-induced astigmatism) and the length (**A**), width (**B**), area (**C**), and cross-sectional area (**D**) of the pterygium.

Pearson correlation and linear regression analyses showed significant correlations between PIA and the pterygium length, width, area, height and cross-sectional area (Fig. 4) (r^2^ = 0.53, 0.42, 0.56, and 0.37, respectively, and all with P values <0.001). No correlation was found between PIA and pterygium height (P = 0.274). After stepwise linear regression, only the pterygium area (P < 0.001) and cross-sectional area (P = 0.018) were independent predictive factors of PIA (Table 2). The following regression model was generated:

**Table 2:** Multivariate Analysis of Potential Predictive Factors for PIA.

Parameters	Regression Coefficient (95% CI*)	P^†^
Pterygium length (mm)	0.58 (−0.96 to 2.12)	0.46
Pterygium width (mm)	0.07 (−1.11 to 1.26)	0.90
Pterygium area (mm^2^)	0.43 (0.30 to 0.57)	<0.001***
Pterygium cross-sectional area (mm^2^)	1.25 (0.22 to 2.28)	0.018*
Pterygium height (μm)	0.00 (−0.01 to 0.01)	0.80

PIA: pterygium induced astigmatism.

*CI: Coefficient interval.

^†^Multiple regression analysis.

Finally, Multiple regression model for PIA was generated as follows:

PIA = 0.43* Pterygium area (mm^2^) + 1.25*Pterygium cross section area (mm^2^) − 2.49 (r^2^ = 0.576, F = 55.33, P < 0.001).

PIA = 0.43* pterygium area (mm^2^) + 1.25*pterygium cross-sectional area (mm^2^) − 2.49 (r^2^ = 0.576, F = 55.33, P < 0.001).

**Figure 5:**
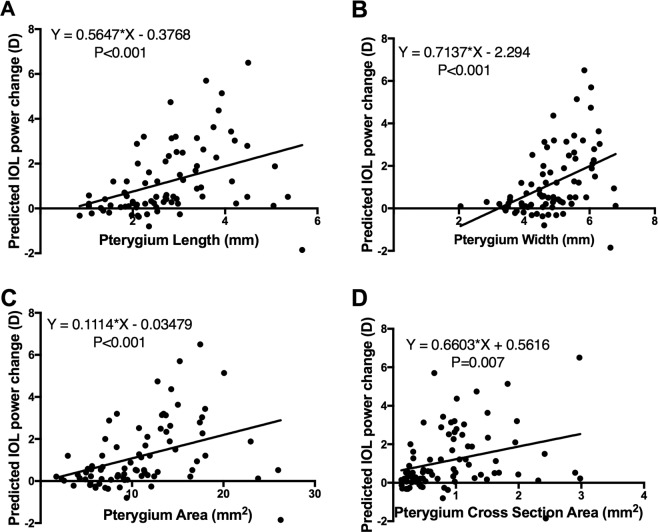
Scatter plots showing the univariate analysis results. Pearson correlation analysis showed significant correlations between the predicted IOL power change (preop.–postop. predicted IOL power)) and the length (**A**), width **(B**), area (**C**), and cross-sectional area (**D**) of the pterygium.

Significant correlations were also found between the pterygium length, width, area and cross-sectional area (Fig. 5) and the predicted IOL power change (r^2^ = 0.15, 0.18, 0.15, and 0.09, respectively, all P values <0.05). No correlation was found between the predicted IOL power change and pterygium height (P = 0.274). After stepwise linear regression, only the pterygium width was an independent predictive factor of the predicted IOL power change (P < 0.001, Table 3). We The following regression model was generated:

**Table 3:** Multivariate Analysis of Potential Predictive Factors for IOL power change (preop.–postop. IOL power).

Parameters	Regression Coefficient (95% CI*)	P^†^
Pterygium length (mm)	0.12 (−0.39 to 0.63)	0.644
Pterygium width (mm)	0.51 (0.10 to 0.91)	0.015*
Pterygium area (mm^2^)	−0.03 (−0.23 to 0.18)	0.784
Pterygium cross-sectional area (mm^2^)	−0.22 (−0.89 to 0.45)	0.507
Pterygium height (μm)	0.002 (−0.002 to 0.006)	0.272

*CI: Coefficient interval.

^†^Multiple regression analysis.

Finally, Multiple regression model for IOL power change was generated as follows:

IOL power change = 0.51*Pterygium width (mm) − 2.42 (r^2^ = 0.205, F = 10.08, P < 0.001).

predicted IOL power change = 0.51* pterygium width (mm) − 2.42 (r^2^ = 0.205, F = 10.08, P < 0.001).

## Discussion

We conducted a three-dimensional evaluation of the pterygium and investigated the effects of its invasion cross-sectional area on the corneal astigmatism and IOL power calculation. We found that the presence of pterygium could cause a significant increase in the predicted IOL power. The pterygium surface area and cross-sectional area were associated with PIA, while only the pterygium width was associated with the predicted IOL power change. To the best of our knowledge, this study may be the first to take the pterygium invasion cross-sectional area, a neglected parameter which is very important, into consideration to study the effects of a pterygium.

Corneal astigmatism induced by a pterygium has been investigated by several studies^5,7,10–12^. Tomidokoro *et al*.^5^ found a correlation between the percentage of pterygium extension onto the cornea (%) and induced corneal astigmatism. Mohammad-Salih *et al*.^7^, Kim *et al*.^10^ and Han *et al*.^13^ paid attention to the pterygium length, width and area and found that PIA was associated with these parameters. Our study found out that the pterygium surface area was an independent predictor of PIA (P < 0.001), which was in consistency with these previous studies. In addition, we also found that the cross-sectional area of the pterygium invading the corneal stroma was another independent predictor of PIA. Contractile elements and fibrous connective tissue within the pterygium were previously assumed to exert traction force and flatten the cornea mechanically^5,12,14,15^; this assumption was verified by our study, because the TCRP increased significantly after surgery, meaning that pterygia could flatten the cornea. Therefore, based on the above findings, we hypothesized that the mechanical force of the pterygium might increase with its surface size and invasion cross-sectional area, resembling the stronger the tree was, the deeper and firmer their roots could attach. This scenario resulted in deepening of the cornea at the horizontal meridian and consequently more severe PIA.

Our study also showed that the presence of pterygium increased the predicted IOL power. The mean predicted IOL power decrease after pterygium surgery was 1.17 D, and a predicted IOL power change ≥1 D was found in 40.74% of the eyes. Kamiya *et al*.^8^ investigated the refractive outcomes of simultaneous pterygium excision and cataract surgery and found myopic refractive errors after surgery were associated with the pterygium length. Koc *et al*.^9^ further demonstrated that the IOL power change was significantly correlated with the pterygium length and area using univariate analysis. They found that with a horizontal length of 2.5 to 4.0 mm and an area of 5.5 to 8.0 mm^2^, the implanted IOL power should be at least 0.50 D smaller than the calculated IOL power. Our result confirmed the findings of previous studies. In addition, using stepwise linear regression, we evaluated the influences of four pterygium parameters and found that only the pterygium width was an independent predictor of the predicted IOL power change. Besides, we also found that the predicted IOL error was >0.5 D if the pterygium width was ≥4 mm. Moreover, the predicted IOL power would increase by 0.7 D along with every 1 mm increase in the pterygium width. Meanwhile, the average predicted IOL power change in our study (1.17 D) was more significant than that of Koc *et al*.’s^9^ (0.59 D). We assumed the difference was attributable to methodology and sample discrepancies.

So, for patients with a concurrent cataract and primary pterygium, what’s the best surgical strategy? Combined cataract and pterygium surgery was convenient but might reduce severe IOL power error after surgery. From our study, PIA was mainly affected by pterygium area and invasion cross-sectional area while the predicted IOL power change was mainly affected by the pterygium width. Thus, we recommend that surgeons comprehensively take the PIA and predicted IOL power change into consideration. For the large, wide or deep invades pterygium, pterygium surgery is strongly suggested separately before cataract surgery rather than combined surgery.

In this study, we only included primary pterygium, because the corneal biomechanical alterations of a recurrent pterygium are more complicated than those of the primary pterygium due to the prior surgery.

In conclusion, the presence of a pterygium induces significant corneal astigmatism, flattens the cornea, and increases the predicted IOL power. The PIA is influenced by its area and cross-sectional area invading the corneal stroma, while the predicted IOL power change is affected by the pterygium width. Cataract surgeons can evaluate the effects of a pterygium according to its three-dimensional parameters and prepare an optimal surgical strategy for patients with a concurrent pterygium and cataract.
